# Pancreatic duct obstruction after pancreaticojejunostomy: implications for early prediction and prevention of long-term pancreatic complications

**DOI:** 10.1186/s12876-018-0777-z

**Published:** 2018-04-23

**Authors:** Lingfu Zhang, Dianrong Xiu, Chunhui Yuan, Bin Jiang, Zhaolai Ma

**Affiliations:** Department of General Surgery, Peking University Third Hospital, Beijing, 100191 China

**Keywords:** Pancreaticojejunostomy, Pancreatic duct obstruction, Postoperative long-term pancreatic complication

## Abstract

**Background:**

Pancreatic duct obstructions are common in patients with pancreaticoduodenectomy. However, it is often neglected in follow up. This study was to review the outcomes of pancreatic duct obstruction and explore the prevention of pancreatic duct obstruction.

**Methods:**

A retrospective analysis of 78 patients undergoing pancreaticojejunostomy without reccurence of disease within 24 months between 2004 and 2014. Pancreatic duct obstruction and long-term pancreatic complications were analysed.

**Results:**

Twenty-five patients developed pancreatic duct obstruction following pancreaticojejunostomy, 13 of whom were found to have long-term pancreatic complications. The presence of pancreatic duct obstruction and early pancreatic obstruction were associated with long-term pancreatic complications, respectively (*p* = 0.002, *p* = 0.002). There are 10 patients with pancreatic duct stent more than 24 months, the postoperative median pancreatic parenchymal thickness in these 10 patients (17.1 mm, range 8.0 to 24.7 mm) was not significantly change than the median in them preoperative (16.4 mm, range 7.2 to 24.7 mm; *p* = 0.747). All of them have no long-term pancreatic complications, though the difference was not significantly (*p* = 0.068).

**Conclusions:**

Early pancreatic duct obstruction is associated with postoperative pancreatic long-term complications. Sustained internal pancreatic stent may improve pancreatic duct obstruction.

## Background

Pancreaticoduodenectomy (PD) has been established as an indication for periampullary lesions. Recent progress in surgical techniques and postoperative management have improved morbidity and motality of patients treated with PD [1]. On the contrary, long-term pancreatic complications such as endocrine and exocrine insufficiency have become important problems in the late postoperative period for patients with PD [2–4].

In fact, little is known about the course of pancreatic endocrine and exocrine insufficiency after resection of pancreatic tumors [5, 6], and the diagnosis of exocrine function is either complex or lack specificity [7]. Some studies of long-term results for patients with PD revealed that pancreatic endocrine and exocrine insufficiency may due to postoperative pancreatic duct obstruction, suggesting that the concern of postoperative pancreatic duct obstruction is essential [4, 8, 9]. In addition to endocrine and exocrine insufficiency, postoperative pancreatic duct obstruction may also cause pain, pancreatic duct stone and acute pancreatitis [10–13]. However, little has been investigated on early prediction and prevention of long-term pancreaticojejunostomy complications.

So we aim to report the incidence of postoperative pancreatic duct obstruction, analyze if early pancreatic duct obstruction predicts long-term pancreatic complications and explore whether sustained pancreatic stent tube prevent pancreatic duct obstruction in patients who underwent PD or segment resection of pancreas with end-to-end invaginated pancreaticojejunostomy.

## Methods

### Study patients

With approval of the Clinical Ethics Committee of Peking University Third Hospital, the patients’ data were analyzed anonymously because written consent was not obtained from all participants. Pancreaticoduodenectomy or segment resection of pancrea with invaginated end-to-end panceaticojejunostomy was performed for pancreatic, periampullary, biliary, or duodenal diseases in 341 patients between January 2004 and December 2014. Seventy-eight of them consisting of the following characteristics were enrolled in this study. They all had no relapse within 24 months and received a continued pancreas protocol computed tomography (CT) at our institution. All the CT data are store and analyzed in our picture archiving and communication system (PACS). Similar procedures were performed by a single team of experienced pancreatic surgeons. All patients underwent end-to-end invaginated pancreaticojejunostomy for reconstruction of the pancreatic stump by the same surgeon. All pancreaticojejunostomy consisted of two-layer sutures, the inner layer anastomosis was performed in a capsule-to-full-thickness fashion and the outer layer anastomosis in a capsule-to-seromuscular fashion after the main pancreatic duct inserted by a matching pancreatic stent tube. Specially, in our centre, for patients with suspected pathology of pancreatic ductal adenocarcinoma (PDAC) during operation, I_125_ radioactive seeds were implanted in the area surrounded superior mesenteric artery (SMA) in 3 patients.

### Definition and treatment of long-term pancreatic complications

#### Assessment of long-term pancreatic function

To assess new-onset pancreatic exocrine insufficiency, the presence of steatorrhea was asked in clinics. Clinical steatorrhea was defined by more than 3 times pasty or greasy stool per day after fatty meals or it is necessary to take pancreatic enzyme to solve their symptoms associated with dyspepsia. Patients with pancreatic exocrine insufficiency before the operation were excluded during the analysis for new-onset pancreatic exocrine insufficiency. The endocrine function was defined by fasting blood glucose level and glycohemoglobin A1C (HbA1c) without administration of an oral hypoglycemic agent or insulin. A diagnosis of diabetes mellitus was made based on the criteria set by the 1985 World Health Organization study group on diabetes mellitus [11]. Patients with diabetes diagnosed before the operation were excluded during the analysis for new-onset diabetes.

#### Assessment of pancreatic obstruction associated pain or pancreatitis

Recurrence of abdominal pain and pancreatitis (Episodic epigastric and back pain; biochemical pancreatitis) was defined as pancreatic obstruction associated pain or pancreatitis.

#### Treatment of patients with long-term pancreatic complications

Patient with recurrent abdominal pain and pancreatitis underwent endoscopic treatment or long-acting octreotide. Patients with pancreatic exocrine insufficiency were given daily pancreatic enzyme replacement therapy and new-onset diabetes given insulin or oral hypoglycemic drugs.

### Morphological changes of the remnant pancreas and definition of pancreatic duct obstruction

**Fig. 1:**
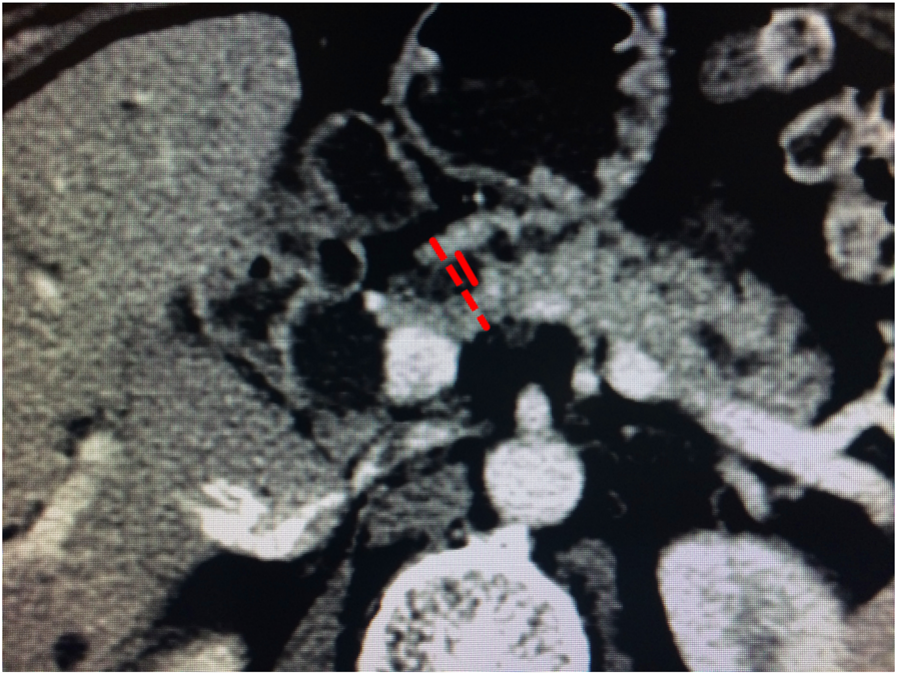
Diameter of the main pancreatic duct (MPD) (D) and thickness of the pancreas (T) were measured on enhanced computed tomography images. Preoperatively, the values were measured along the line at the left edge of the superior mesenteric vein (SMV). Postoperatively, the parameters were measured at the same area in the remnant pancreas. The degree of pancreatic atrophy was calculated by the results of T – D. Note: Dotted line indicates the thickness of the pancreas; continuous line indicates diameter of the main pancreatic duct

Preoperative and postoperative pancreatic remnant changes were evaluated by computed tomography (CT) images at our institution PACS. Diameter of the main pancreatic duct (MPD) and thickness of the pancreas were measured on enhanced computed tomography images (Fig. 1). Pancreatic duct obstruction was defined by the MPD ≥3 mm. The patients were divided into two groups based on the dilation of main pancreatic duct. Parenchymal thickness was calculated by substracting the pancreatic duct diameter from the total gland thickness.

Especially, early pancreatic duct obstruction is evaluated in patients with postoperative CT pancreatic remnant changes within 12 months after operation. Pancreatic atrophy is defined by obvious remnant parenchymal thickness decline (Thickness decline ≥1/2).

### Statistical analysis

We analyzed the perioperative factors in all 78 patients to identify the risk factors of long-term pancreatic complications after end-to-end invaginated pancreaticojejunostomy. The pancreatic duct diameters were expressed as mean ± standard deviation or medians and ranges. The independent two sample *t* test was used for continuous data. Categorical data were compared by a chi-square test or Fisher’s test when necessary. Multivariate analysis was performed using multiple logistic regression models. A *P* value < 0.05 was considered statistically significant. Values of *p* < 0.05 were considered statistically significant, and odds ratios for multivariate analysis were reported with corresponding 95% confidence intervals. The statistical analyses were performed with version 21.0 SPSS software (SPSS, Chicago, IL, USA).

## Results

Among the 78 patients with pancreaticojejunostomy, 73 patients underwent PD and 5 patients segment resection of pancreas, and 63 of them had CT data evaluating early pancreatic duct obstruction that was recorded in our PACS.

Based on the criteria of MPD ≥ 3 mm, there are 32 patients with preoperative pancreatic duct obstruction and 7 patients with preoperative long-term pancreatic complications, the difference is significant between preoperative pancreatic duct obstruction and preoperative long-term pancreatic complications (*p* = 0.038).

### Demographics, postoperative long-term pancreatic complications occurrence, and predictors of postoperative long-term pancreatic complications

The mean age of the 78 patients was 56 years (range 14 to 78 years), and 47 of the 78 (60%) patients were male. Seventy-three patients (94%) underwent PD, compared with 5 (6%) who underwent segment resection of pancreas with invaginated end-to-end panceaticojejunostomy for the distal pancreatic stump. Thirty-five (45%) patients were benign disases.

Long-term pancreatic complications occurred in 18 (23%) patients, including 2 patients with preoperative exocrine insufficency, 5 patients with concomitant complications, consisting of 11 new on-set pancreatic exocrine insufficiency, 4 new on-set diabetes and 8 recurrent pain/pancreatitis (Table 1). Among them, the median interval between the operation and the occurrence of postoperative complications are 24 months (range 8 to 36 months).

**Table 1:** Patients with postoperative new-onset long-term pancreatic complications: Types of long-term pancreatic complications and its association with postoperative pancreatic duct obstruction

Long-term complications	Pancreatic duct obstruction (*n* = 25)	No pancreatic duct obstruction (*n* = 53)	*P* value
Exocrine insufficiency, n (%)	7 (28)	4 (8)	0.032
Endocrine insufficiency, n (%)	3 (12)	1 (2)	0.094
Pain and pancreatitis, n (%)	7 (28)	1 (2)	0.001

In evaluating predicators for long-term pancreatic complications, univariate analysis revealed the following factors were associated with long-term pancreatic complications: postoperative pancreatic duct diameter ≥ 3 mm (*p* < 0.001), postoperative pancreatic parenchymal atrophy (*p* < 0.001), and adjuvant therapy (*p* = 0.002). There was no statistically significant relationship between all other variables and long-term pancreatic complications, including pathology, pancreatic fistula, type of operation (PD or segment pancreatic resection), I_125_ radioactive seeds implantation and pancreatic duct stent retained>2 years. On multivariate analysis, postoperative pancreatic duct diameter ≥ 3 mm (*p* = 0.002) and postoperative pancreatic parenchymal atrophy (*p* = 0.001) were remained the significant predictor of long-term pancreatic complications (Table 2).

**Table 2:** Predictors of long-term pancreatic complications for patients with documented pancreatic duct diameter after operation

Variable	Long-term complication rate	Univariate *p* value	Multivariate p value	OR (95% CI)
Preoperative pancreatic duct diameter ≧ 3mm, n (%)				
No	10/42 (23.8%)	0.777		
Yes	6/32 (18.8%)			
Postoperative pancreatic duct diameter ≧ 3mm, n (%)				
No	5/53 (9.4%)	< 0.001	0.002	19.0 (3.022-118.984)
Yes	13/25 (52.0%)			
Postoperative pancreatic parenchymal atrophy, n (%)			0.001	21.2 (3.324-135.697)
No	5/56 (8.9%)	< 0.001		
Yes	13/22 (59.1%)			
Pathology, n (%)				
Benign	12/35 (34.3%)	0.057	0.253	0.4 (0.067-2.038)
Malignant	6/43 (14.0%)			
Type of operation, n (%)				
Pancreaticoduodenectomy	16/73 (21.9%)	0.326		
Segment resection of pancreas	2/5 (40.0%)			
Pancreatic fistula, n (%)				
No	15/61 (24.6%)	0.748		
Yes	3/17 (17.6%)			
Adjuvant therapy, n (%)				
No	13/59 (22.0%)	0.002	0.087	9.3(0.725-118.506)
Chemotherapy	0/12 (0.0%)			
long-acting octreotide	5/7 (71.4%)			
I_125_ radioactive seeds implantation, n (%)				
No	18/74 (24.3%)	1.000		
Yes	0/3 (0.0%)			
Pancreatic duct stent retained > 2 years, n (%)				
No	18/68 (26.5%)	0.068		
Yes	0/10 (0.0%)			

### Correlation between early pancreatic duct obstruction and long-term pancreatic complications

Among the 63 patients with early pancreatic duct diameter data, 46 of them have not present preoperative pancreatic duct obstruction, 9 of whom develop pancreatic duct obstruction within 12 months (14 patients develop pancreatic duct obstruction in the final follow up), and only one pancreatic duct obstruction recovered in the follow up, so we infer that pancreatic duct obstruction occurred early after operation and the spontaneous obstruction relief is rare. Among the 63 patients, there are 13 patients with postoperative long-term pancreatic complications in the follow up. Further analysis demonstrated that the median pancreatic duct diameter in the 13 patients with long-term pancreatic complications (4.1 mm, range 1.3 to 7.0 mm) was significantly higher than the median in those without (1.9 mm, range 1.0 to 10.0 mm; *p* = 0.002). The early pancreatic duct obstruction correlated well with postoperative long-term pancreatic complications (*p* = 0.002).

### Patient demographics and perioperative variables for patients with and without postoperative pancreatic duct obstruction

The 2 groups (patients who had postoperative pancreatic duct obstruction vs those who did not) were similar with respect to patient demographics and perioperative variables (Table 3). There was no significant difference in patient age or sex, preoperative pancreatic duct diameter, type of operation, I_125_ radioactive seeds implantation, pancreatic fistula, adjuvant therapy and pancreatic duct stent retained more than 2 years. The only variable found to be statistically different between the 2 groups was postoperative pancreatic parenchymal thickness, 9.5 ± 4.1 mm compared with 14.1 ± 4.9 mm (*p* < 0.001).

**Table 3:** Correlation of postoperative pancreatic duct obstruction with remnant pancreatic morphological changes and clinicopathologic characteristics in patients who underwent pancreatic resection and pancreaticojejunostomy

Variable	Pancreatic duct obstruction after pancreaticojejunostomy		*P* value
	Present (*n* = 25)	Absent (*n* = 53)	
Mean age at operation, y	54.6±16.1 (range -)	56.7±16.1 (range 14-79)	0.712
Sex, M:F (%)	17 (68):8 (32)	30 (57):23 (43)	0.458
Pancreatic duct diameter (mm)			
Before operation			0.153
Mean ± SD	3.5 ± 2.4	3.0 ± 1.9	
Median [range]	2.5 [1.0-10.0]	2.8 [1.0-9.0]	
After operation			0.000
Mean ± SD	5.7 ± 2.6	1.8 ± 0.6	
Median [range]	[3.3-13.0]	[1.0-3.0]	
Pancreatic parenchymal thickness (mm)			
Before operation			0.870
Mean ± SD	14.0 ± 4.9	14.3 ± 5.7	
Median [range]	18 [10.7-25.0]	17.4 [4.5-30.0]	
After operation			
Mean ± SD	9.5 ± 4.1	14.1 ± 4.9	0.000
Median [range]	11 [2.5-16.7]	14.7 [3.0-28.3]	
Type of operation, n (%)			1.000
Pancreaticoduodenectomy	24	49	
Segment resection of pancreas^*^	1	4	
Pancreatic duct stent retained > 2 years, n (%)	1 (4)	9 (17)	0.155
Adjuvant therapy, n (%)	9 (36)	11 (21)	0.397
I_125_ radioactive seeds implantation, n (%)	2 (8)	1 (2)	0.245
Pancreatic fistula, n (%)	3 (12)	14 (26)	0.240
Malignant tumor, n (%)	13 (52)	30 (57)	0.809
Follow-up period (mo)			0.551
Mean ± SD	39.8 ± 20.4	37.1 ± 22.0	

*Segment resection of pancreas: Medial pancreatectomy without duodenectomy and the distal pancreatic stump was covered with invaginated end-to-end panceaticojejunostomy.

### Treatment of patients with long-term pancreatic complications

One patient with recurrent abdominal pain and pancreatitis underwent endoscopic treatment, pancreatic duct stricture was detected and an internal pancreatic stent tube was placed in the pancreatic duct, the symptom was relieved after that. The other remaining 7 patients with symptom relieved after long-acting octreotide therapy or octreotide combined with other medicine. Eleven patients with pancreatic exocrine insufficiency were given daily pancreatic enzyme replacement therapy and 4 patients with new-onset diabetes were given insulin or oral hypoglycemic drugs.

### The characteristics of 10 patients with pancreatic duct stent more than 24 months

So far, there are 17 patients have sustained internal stent tube, with a median interval of 24 months (range 12 to 120 months), while in the remaining 61 patients with internal stent tube fall off, when 3, 6, 12, 24 months serve as the checklist reference, 28 patients have available fall off time interval during the previous checklist reference, and 24 of them lost their stent tube within 6 months.

Considering that all 10 patients with pancreatic duct stent more than 24 months have no long-term pancreatic complications, though the difference was not significantly compared with the group with no pancreatic duct stent more than 24 months (*p* = 0.068). A further analysis was conducted to evaluate the feasibility of sustained pancreatic duct stent in preventing long-term pancreatic complications. The postoperative median pancreatic duct diameter in the 10 patients with pancreatic duct stent more than 24 months (2.0 mm, range 1.0 to 4.3 mm) was lower than the median in them preoperative (2.2 mm, range 1.1 to 7.0 mm). Furthermore, the postoperative median pancreatic parenchymal thickness in the 10 patients with pancreatic duct stent more than 24 months (17.1 mm, range 8.0 to 24.7 mm) was not significantly change than the median in them preoperative (16.4 mm, range 7.2 to 24.7 mm; *p* = 0.747).

## Discussion

This work presents early postoperative pancreatic duct obstruction as a potentially simple tool by which the pancreatic surgeon may predict and ahead of time treat their patients following pancreaticojejunostomy with long-term pancreatic complications. Furthermore, presumes that sustained internal pancreatic duct stent may prevent the occurrence of pancreatic duct obstruction.

First, postoperative pancreatic duct obstruction is an indicator for postoperative long-term pancreatic complications. Our results demonstrate that the statistical difference is significant between pancreatic duct obstruction and long-term pancreatic complications, regardless preoperatively (*p* = 0.038) or postoperatively (*p* = 0.002). This result is in accordance with previous studies [4, 8, 9], which speculated that pancreatic duct obstruction maybe a cause of long-term pancreatic complications including exocrine and endocrine insufficency. Although the overall exocrine pancreatic function is also influenced by the pancreatic stimulus, more attention has been paid on the pancreatic remnant reserve and pancreatic duct outflow [5, 9, 14, 15]. In our study cohort, there were 5 patients underwent segment resection of pancreas, the statistical difference is no significant compared with patients underwent pancreaticoduodenectomy in long-term pancreatic complications (*p* = 0.326). But postoperative pancreatic duct obstruction and postoperative pancreatic remnant atrophy are statistically different in patients with or without long-term pancreatic complications (*p* = 0.002, *p* = 0.001). Those results seem to support the importance of remnant reserve and pancreatic duct outflow in pancreatic function. There is no definite evidence proves that pancreatic duct obstruction is a cause of remnant reserve. Our results exhibited that pancreatic duct obstruction associated with pancreatic atrophy (p = 0.001). So, postoperative pancreatic duct obstruction may cause or at least partially postoperative long-term pancreatic complications.

Second, considering most patients develop pancreatic duct obstruction within 12 month (9/14), and only one pancreatic duct obstruction recovered in the follow up, so we infer that pancreatic duct obstruction occurred early after operation and the spontaneous obstruction relief is rare. So, based on the previous results, we inferred that early pancreatic duct obstruction may predict long-term pancreatic complications after pancreaticojejunostomy. In our 64 patients with postoperative CT pancreatic remnant changes within 12 months after operation, patients with early pancreatic duct obstruction tend to develop long-term pancreatic complications (*p* = 0.001). Throughout the past decade, several studies have explored the use of pancreatic duct obstruction to predict long-term pancreatic complications after pancreaticoduodenectomy [4, 8–11]. Although methods differ between studies, and only one author has evaluated early pancreatic duct obstruction is a predictor for long-term pancreatic complications [4], these results indicated that early pancreatic duct obstruction may serve as a simple tool in predicting postoperative long-term pancreatic obstruction.

Third, duration of the internal pancreatic stent may protect the pancreatic duct obstruction. As we have demonstrate that pancreatic duct obstruction may cause pancreatic remnant atrophy and following long-term pancreatic complications, so we presume that preventing pancreatic duct obstruction is likely to decrease the rate of long-term pancreatic complications. In our study group, there are 10 patients with internal pancreatic stent more than 24 months, the postoperative pancreatic parenchymal thickness in these 10 patients was not significantly change than preoperative (*p* = 0.747). And these patients have no long-term pancreatic complications, though the difference was not significantly compared with the group with no pancreatic duct stent more than 24 months (*p* = 0.068). So, we assumed that the internal pancreatic stent for pancreatic duct might offer a potential deduction of pancreatic duct obstruction and following long-term pancreatic complications. Notwithstanding the data from Murakami M demonstrated that [4] pancreatic duct tube seems to be ineffective for resolving anastomotic stricture, their studies did not present the duration of the pancreatic duct tube, and since 2015 we have taken nonabsorbable suture material in fixation of internal pancreatic duct stent tube in our center, further outcomes will confirm the feasibility of sustained internal pancreatic stent tube in preventing of long-term pancreatic complication.

There are some limitations to our study. First, with 78 patients eligible for inclusion, the sample size is relatively small, more data will be needed to validate the results from this study. Second, due to the lack of objective method to evaluate long-term pancreatic complications, we take the relative subjective method to define long-term complications, although they were also taken by other studies [10, 11]. However, there were three different points between our study and previous studies. First, the technical approach in anastomosis of pancreaticojejunostomy, we all took invaginated end-to-end pancreaticojejunostomy, excluding the bias in anastomosis of pancreatic remnant. Second, importantly the metric we investigated, main pancreatic duct thickness on pre- and postoperative CT scans, is readily available and easily translatable to current clinical practice making our results immediately applicable. Third, we assumed that the sustained internal pancreatic stent tube for pancreatic duct might offer a potential deduction of pancreatic duct obstruction and following long-term pancreatic complications.

## Conclusions

In conclusion, the incidence of pancreatic duct obstruction after PD with end-to-end invaginated pancreaticojejunostomy is common. This study serves as the first to date demonstrating the relationship between pancreatic duct obstruciton and long-term complications after PD with end-to-end invaginated pancreaticojejunostomy. Moreover, this study demonstrates that most pancreatic duct obstruction occurs within 12 months. Therefore, early pancreatic duct obstruction is a simple tool to screen high risk patients with the tendency to develop long-term pancreatic complications. Furthermore, sustained internal pancreatic duct stent may prevent the occurrence of pancreatic duct obstruction. Although the studies conducted so far have included low numbers of patients and have been unable to draw firm conclusions.

## Abbreviations

CT: computed tomography
HbA1c: glycohemoglobin A1C
MPD: main pancreatic duct
PACS: picture archiving and communication system
PD: Pancreaticoduodenectomy
PDAC: pancreatic ductal adenocarcinoma
SMA: superior mesenteric artery
